# The Anti-*Candida albicans* Agent 4-AN Inhibits Multiple Protein Kinases

**DOI:** 10.3390/molecules24010153

**Published:** 2019-01-02

**Authors:** Maciej Masłyk, Monika Janeczko, Aleksandra Martyna, Sławomir Czernik, Małgorzata Tokarska-Rodak, Marta Chwedczuk, Béatrice Foll-Josselin, Sandrine Ruchaud, Stéphane Bach, Oleg M. Demchuk, Konrad Kubiński

**Affiliations:** 1 Department of Molecular Biology, Institute of Biotechnology, The John Paul II Catholic University of Lublin, ul. Konstantynów 1i, 20-708 Lublin, Poland; maciekm@kul.pl (M.M.); mjanec@kul.pl (M.J.); aleksandra.martyna@kul.pl (A.M.); 2 Innovation Research Centre, Pope John Paul II State School of Higher Education in Biala Podlaska, Sidorska 95/97, 21-500 Biala Podlaska, Poland; czernikslawomir@gmail.com (S.C.); m.chwedczuk@pswbp.pl (M.C.); 3 Institute of Health Sciences, Pope John Paul II State School of Higher Education in Biala Podlaska, Sidorska 95/97, 21-500 Biala Podlaska, Poland; rodak.malgorzata@gmail.com; 4 Sorbonne Université, USR3151 CNRS/UPMC, Plateforme de criblage KISSf (Kinase Inhibitor Specialized Screening Facility), Station Biologique, Place Georges Teissier, F-29688 Roscoff, France; beatrice.josselin@sb-roscoff.fr (B.F.-J.); sandrine.ruchaud@sb-roscoff.fr (S.R.); bach@sb-roscoff.fr (S.B.); 5 Pharmaceutical Research Institute, Rydygiera Street 8, 01-793 Warsaw, Poland; O.Demchuk@IFarm.eu

**Keywords:** *Candida albicans*, arylcyanomethylenequinones, 4-AN, protein kinases, antifungal agent, atomic force microscopy

## Abstract

Small molecules containing quinone and/or oxime moieties have been found as promising anti-fungal agents. One of them is 4-AN, a recently reported potent anti-*Candida* compound, which inhibits the formation of hyphae, decreases the level of cellular phosphoproteome, and finally shows no toxicity towards human erythrocytes and zebrafish embryos. Here, further research on 4-AN is presented. The results revealed that the compound: (i) Kills *Candida* clinical isolates, including these with developed antibiotic resistance, (ii) affects mature biofilm, and (iii) moderately disrupts membrane permeability. Atomic force microscopy studies revealed a slight influence of 4-AN on the cell surface architecture. 4-AN was also shown to inhibit multiple various protein kinases, a characteristic shared by most of the ATP-competitive inhibitors. The presented compound can be used in novel strategies in the fight against candidiasis, and reversible protein phosphorylation should be taken into consideration as a target in designing these strategies.

## 1. Introduction

**Figure 1:**
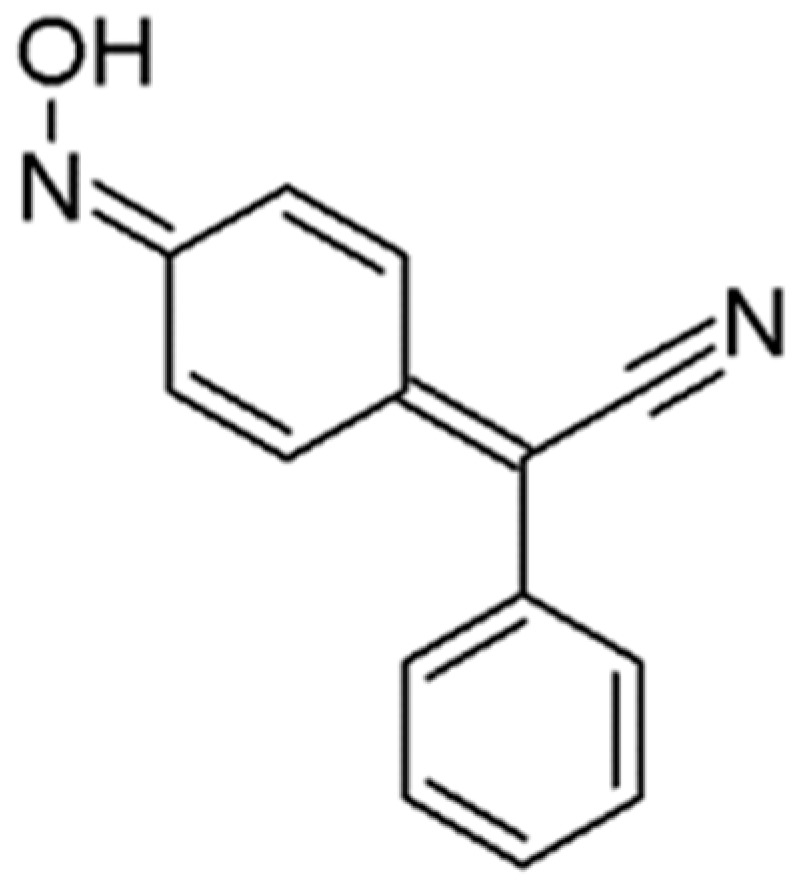
Chemical structure of 4-AN.

*Candida* species cause a majority of fungal infections and significantly contribute to morbidity and mortality worldwide, which makes them a serious threat to public health [1]. Among them, *Candida albicans* followed by *Candida glabrata* appear to be the most pathogenic species [2]. Due to their ability to develop resistance to antifungal drugs, these opportunistic fungi evade therapeutic strategies, and thus they are a serious problem for the treatment and management of *Candida* infections [3]. Among the available antifungal agents, there are azoles (e.g., Fluconazole), polyenes (Amphotericin B), echinocandins (Caspofungin), nucleoside analogs (Flucytosine), and allyamines (Terbinafine). The chemicals exhibit different mechanisms of action in *Candida* cells [4]. Azoles interfere with the synthesis of a constituent of fungal cell membranes ergosterol, while polyenes bind with ergosterol and disturb cell membrane integrity. In turn, echinocandins inhibit the enzyme (1→3)-β-d-glucan synthase and thereby ruin the integrity of the fungal cell wall. Nucleoside analogs stop DNA polymerase action in fungal cells through incorporation into growing DNA strands. Finally, allyamines inhibit squalene epoxidase and thus disturb ergosterol synthesis [4]. Nevertheless, *Candida* cells develop different mechanisms of multidrug resistance against a majority of systemic antifungal drugs. For this reason, there is an urgent need to search for new strategies of fighting against candidiasis. One of such approaches is to synthesize new biologically active antifungal compounds. Compounds that arise from a quinone moiety containing an oxime group are reasonable alternatives. There are many reports on the antimycotic properties of both quinone derivatives and compounds substituted with oximes, including anti-*Candida* activity [5,6,7,8]. Very recently, strong anti-*Candida* activity (MIC = 4 µg/mL) of the arylcyanomethylenequinone oxime (4-AN) (Figure 1) connected with the inhibition of kinase activity has been revealed [9]. There are more than 500 protein kinases in eukaryotic cells that catalyze reversible phosphorylation—a process engaged in almost every aspect of the living cell, including fungi [10]. The phosphorylation status of many proteins is strictly related to *Candida albicans* virulence, usually in such a way that phosphorylation of a given protein strengthens the pathogenic properties of the fungus. This modification can e.g., promote efficient hyphal extension in the Exo84 protein and positively regulate Nap1, a protein that is essential for candidiasis development [11,12]. Therefore, protein kinases can be reasonably considered as molecular targets in newly designed antifungal therapy. The article is a continuation of the recently reported studies on anti-*Candida albicans* activity of the arylcyanomethylenequinone oxime (4-AN) [9]. It has been revealed that the compound inhibits fungal growth with a minimal inhibitory concentration (MIC) value of 4 µg/mL, hyphal formation, and total phosphorylation in *Candida* cells. Additionally, 4-AN showed no evident toxicity against erythrocytes and zebrafish when tested at a concentration of 20 µg/mL (5×MIC). Here, we present the influence of 4-AN on (i) clinical isolates of *Candida*, (ii) fungal biofilm, (iii) cell membrane integrity, (iv) cell and colony morphology studied with atomic force microscopy (AFM). Finally, we tested 4-AN against a panel of 17 protein kinases and performed molecular docking of the compound and the most susceptible enzyme.

## 2. Results and Discussion

### 2.1. Activity of 4-AN Against Clinical Isolates Of Candida and Its Influence on Biofilm and Membrane Permeability

**Figure 2:**
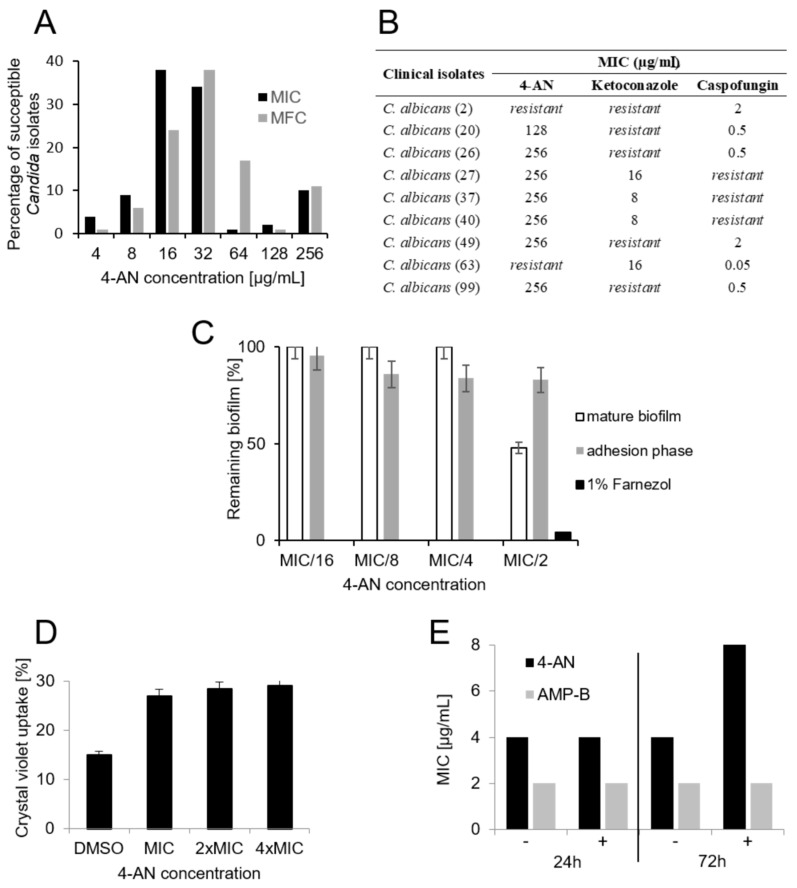
(**A**) Fungicidal and fungistatic effect of 4-AN on clinical Candida isolates. (**B**) Influence of 4-AN on resistant *C. albicans* isolates, (**C**) influence of 4-AN on Candida biofilm. (**D**) Influence of 4-AN on crystal violet uptake by *C. albicans* cells. (**E**) Osmoprotective effect of sorbitol on *C. albicans* cells treated with 4-AN.

As demonstrated in available reports, numerous compounds containing both quinone and oxime moieties show anti-*Candida* activity ranging between 4 and 250 µg/mL [7,13,14]. A vast majority (96%) of the *Candida* clinical isolates tested here were more resistant to 4-AN than the reference strain of *C. albicans* (MIC = 4 µg/mL). The minimal inhibitory concentration values ranged between 4 and 256 µg/mL; however, in the case of 72% of the strains, MICs amounted to 16 or 32 µg/mL (Figure 2A), while the minimal fungicidal concentrations (MFC) were slightly higher, and in the case of 79% of the isolates it ranged between 16 and 64 µg/mL. Among 12 isolates with MIC of 128–256 µg/mL, there are seven strains with developed resistance towards Ketoconazole or Caspofungin (Figure 2B). In 2012, large-scale research on the susceptibility of fungal isolates and reference strains to antifungal systemic drugs was published. Comparison of the antifungal susceptibility profiles of 4240 clinical isolates and 315 reference strains belonging to 53 shared species showed similar results [15]. In this context, our results are promising because 4-AN shows only 4–8-fold lower fungistatic and fungicidal activity against a majority of clinical isolates in comparison with the reference strain. 

It is necessary to mention that because 4-AN concentrations in the neighboring wells were 2-fold different, the results obtained in each replicate were always identical, thus there are no error bars as ± SD or SEM included in the graph presenting MIC and MFC values. One of the fungal invasion stages is biofilm production, which can be strongly related to poor prognosis [16]. While 4-AN did not influence the adhesion phase during biofilm formation, at the concentration of MIC/2 (2 µg/mL) it effectively damaged the mature biofilm structure by 52% (Figure 2C). In comparison with systemic drugs (flucytosine and fluconazole), 4-AN is much more effective in biofilm destruction. Al-Fattani MA and Douglas LJ showed that flucytosine and fluconazole decreased biofilm cell viability by 70% and 30% at 30×MIC and 60×MIC, respectively [17]. 

Crystal violet has been known to penetrate cells with impaired cell membranes and it is a useful tool for the detection of membrane damage [18]. We used the method to verify if 4-AN can alter *Candida* membrane permeability. The obtained results show a slight increase in the crystal violet uptake (12%) by *C. albicans* cells when tested at the MIC concentration (Figure 2D). One of the 1,4-naphtoquinone derivatives tested recently caused higher uptake (31%, [6]) of the dye, which may suggest a different mechanism of 4-AN action that is not directly involved in cell membrane damage.

It is known that the presence of osmoprotectants like sorbitol in culture medium can recover the damaging effects of antimicrobial agents towards the cell membrane [19]. As shown in Figure 2E, 3 days of incubation of cells treated with 4-AN in the presence of sorbitol resulted in a 2-fold increase in the MIC value, while the MIC of the negative control amphotericin B was not changed. This observation may suggest that 4-AN directly or indirectly targets cellular events involved in cell wall synthesis.

### 2.2. Atomic Force Microscopy Studies

*C. albicans* cells can undergo deformation and destruction under the influence of biologically active substances. These changes are the result of disturbances in the nanomechanical properties of cells [20]. The height of the cells and the appearance of their surface consequently change as well. The typical height of *C. albicans* cells is in the range of 1.5 μm–3.5 μm [21] and it changes under the influence of lemon grass oil (700 nm) and lemon grass oil vapor (100 nm) [22]. As shown in the research conducted by Quilès et al., Caspofungin alters and modifies the surface topography, and mechanical changes are observed for susceptible and resistant *C. lusitaniae* strains [23].

**Figure 3:**
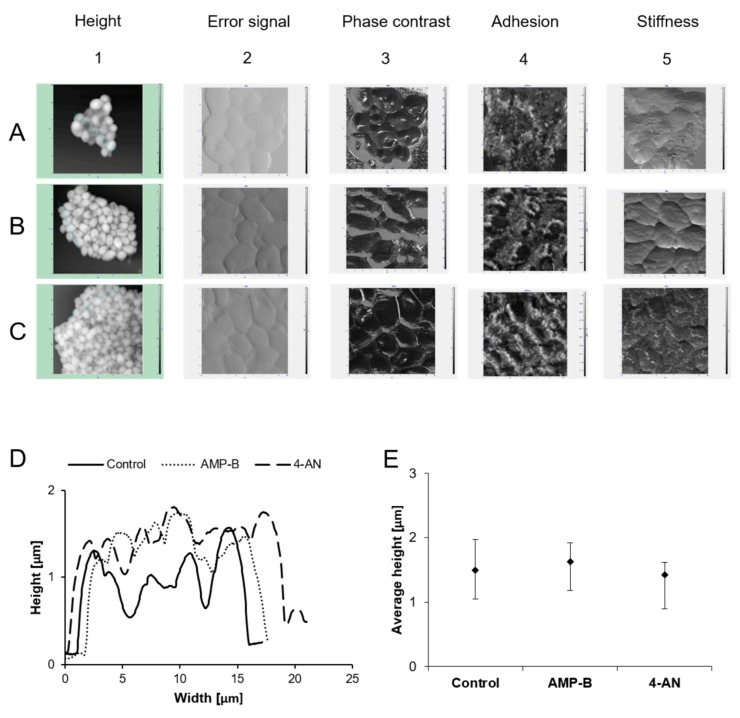
Multiparametric imaging of *Candida albicans* cells by AFM: (**A**) Control cells, (**B**) positive control (AMP-B), and (**C**) cells treated with 4-AN. Cross-sections of images A1, B1, and C1 were traced to determine the height of the cells. Pictorial parameters: height (1), error signal (2), phase contrast (3), adhesion (4), stiffness (5). (**D**) The difference in height between the cells represented by the height profile. (**E**) The height of *C. albicans* cells—standard deviations (SD).

The topography of *Candida albicans* control cells and cells treated with the antifungal compounds AMP-B and 4-AN are shown in Figure 3. The average height of *C. albicans* cells treated with AMP-B is 1.6 μm (min. 1.1 μm–max. 1.9 μm; SD 0.2), which is higher than that of the control cells (1.5 μm; min. 1.0 μm–max. 1.9 μm; SD 0.2) and cells treated with 4-AN (1.4 μm; min. 0.8 μm–max. 1.6 μm; SD 0.2). However, the height difference is not significant when considering SD values. The difference in height between the cells represented by the height profile is illustrated in Figure 3D and E. The surface of *C. albicans* cells became more uneven and rough under the influence of AMP-B (Figure 3(B2)) compared to the control cells (Figure 3(A2)). The surface of *C. albicans* treated with 4-AN (Figure 3(C2)) differs slightly from that of the control cells. Areas with varying softness (force values ranging from 80 to 170 degrees) were observed on the surface of *C. albicans* cells treated with AMP-B (Figure 3(B3)). No such significant differentiation was observed on the surface of the control cells (Figure 3(A3)) and cells treated with 4-AN (Figure 3(C3)). The value of the adhesion force for *C. albicans* cells treated with AMP-B (Figure 3(B4)) was significantly higher (92.6 nN, min. 88.0 nN–max. 108.0 nN; SD 5.7; *p* < 0.001) in comparison with the value obtained for cells treated with 4-AN (Figure 3(C4)) (82.5 nN, min. 78.4 nN–max. 93.1 nN; SD 4.2) and the control cells (Figure 3(A4)) (80.3 nN, min. 75.5 nN–max. 94.4 nN; SD 5.4). The magnitude of cantilever vibration is dependent on sample stiffness (the z-scale shows values ranging from 0 to 8 nA). *Candida* albicans cells treated with AMP-B and 4-AN showed area with differentiated stiffness compared to *C. albicans* control cells.

The presence of areas with varying softness and stiffness as well as higher adhesion force on the surface of *C. albicans* cells treated with AMP-B may indicate a significant destructive effect of AMP-B on the yeast cell wall. It can be assumed that the antifungal compound (AMP-B) has greater destructive properties against *C. albicans* cells than 4-AN.

### 2.3. 4-AN-Mediated Inhibition of Protein Kinases

**Figure 4:**
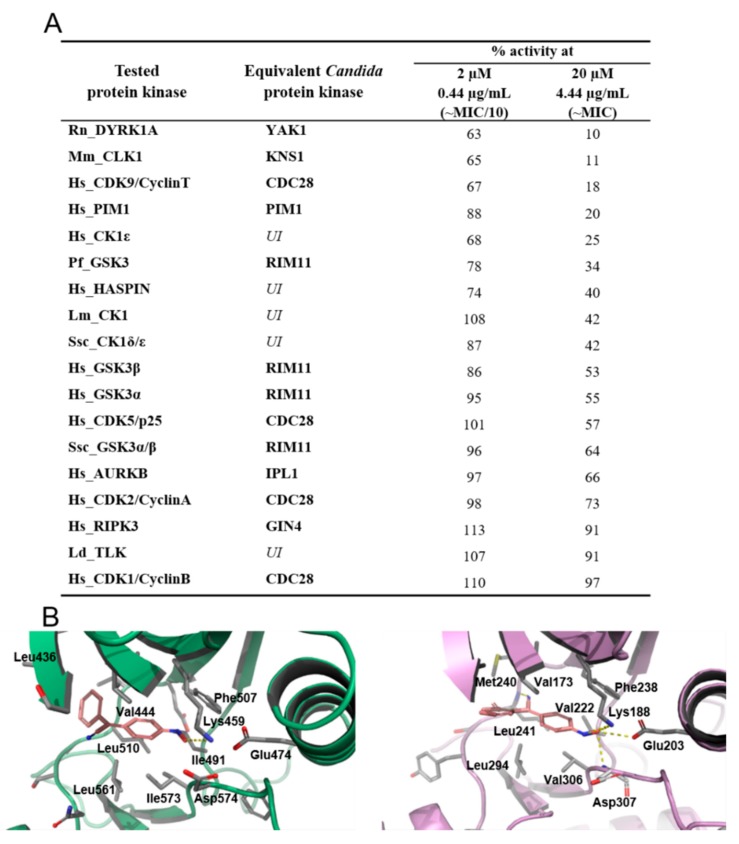
Effect of 4-AN on kinase activity. (**A**) Inhibitory activities of 4-AN against a short panel of disease-related protein kinases. The table displays the remaining kinase activities detected after treatment with 2 or 20 µM of 4-AN. Candida equivalents of the tested protein kinases were derived from the Candida Genome Database (http://www.candidagenome.org/) and ProViz tool (http://proviz.ucd.ie/), UI—Candida equivalent unidentified. Results are expressed in % of maximal activity, i.e., measured in the absence of inhibitor but with an equivalent dose of DMSO (solvent of the tested compounds). ATP concentration used in the kinase assays was 15 µmol/L (values are means, n = 2). Kinases are from human origin unless specified: Rn, *Rattus norvegicus*; Mm, *Mus musculus*; Pf, *Plasmodium falciparum*; Lm, *Leishmania major*; Ssc, *Sus scrofa domesticus*; Ld, *Leishmania donovani*. (**B**) Docked binding modes for 4-AN in the ATP-binding pocket of YAK1 (**left**) and DYRK1A (**right**).

4-AN was tested at concentrations of MIC/10 and MIC against a panel of 17 protein kinases from different organisms. Although *Candida* protein kinases are not available at kinase profiling services, homologues of a given protein kinase from different organisms (from yeast to human) show a high degree of conservation as to sensitivity towards small-molecule inhibitors [24]. The *Candida albicans* equivalents of several studied kinases are listed in Figure 4A. The results revealed the susceptibility of some kinases to the action of the tested compound. At the MIC concentration, 4 kinases belonging to the CMGC (DYRK1A, CLK1, CDK9) and CAMK (PIM1) group were inhibited by at least 80% (Figure 4A). However, other kinases included in the test also showed some sensitivity towards 4-AN (reduction of activity by 75 to 27%). 4-AN is structurally related to already described kinase inhibitors and as expected we previously showed that this compound can reduce the level of phosphoproteome in *C. albicans*, and moderately inhibits protein kinase CK2 [9]. The results presented here suggest that the decrease in cellular phosphorylation is the consequence of inhibition of various kinases and 4-AN acts as a multi-kinase inhibitor.

In order to verify if the compound can occupy the ATP-binding site and interact with key amino acid residues responsible for ligand binding, we used bioinformatics tools to investigate the ability of 4-AN to fit in the active site of the DYRK1A, which is the most affected by the compound, and a model of YAK1, i.e., a homologous kinase from *C. albicans*. Since the 3D crystal structure of YAK1 has not been developed thus far, it was modeled using the SWISS-MODEL tool. Next, AUTODOCK Vina was employed for molecular docking calculations. The analysis of the obtained complexes, 4-AN—DYRK1A and 4-AN—YAK1, revealed that in both cases the compound adopted a similar conformation inside the ATP-binding pocket, suggesting a competitive mode of inhibition (Figure 4B). The oxime group coordinates corresponding Lys459/188, Glu474/203, and Asp574/307 residues forming electrostatic interactions, which is usually found in the case of potent kinase inhibitors [25]. In turn, the aromatic rings of 4-AN occupy the hydrophobic cavity formed by Val444/173, Phe507/238, Leu510/241, Leu561/294, and Ile573/Val306.

It is intriguing that the three *Candida* equivalents of the four most susceptible protein kinases, namely YAK1, KNS1, and PIM1 are involved in cellular events responsible for morphological changes, hyphal induction, and biofilm formation [26,27,28]. This corresponds with our recent and present data revealing that 4-AN can inhibit the formation of hyphae [9] and damage mature biofilm (Figure 2B).

## 3. Materials and Methods 

### 3.1. 4-(Hydroxyimino)cyclohexa-2,5-dien-1-ylidene](phenyl)ethanenitrile (4-AN)

4-(hydroxyimino)cyclohexa-2,5-dien-1-ylidene](phenyl)ethanenitrile (4-AN) was synthesized as previously reported [9,29]. The structure of 4-AN is depicted in Figure 1.

### 3.2. Microbial Strains 

4-AN was screened for its in vitro antifungal activity against the standard strains: *Candida albicans* ATCC 10231, and 69, 20, and 11 clinical isolates of *Candida albicans*, *Candida glabrata*, and *Candida tropicalis*, respectively. The isolates were derived from gynecological patients (vaginal strains) and from sputum. The fungi were identified using VITEK^®^ 2 YST ID cards (Biomérieux).

### 3.3. Determination of Minimal Inhibitory Concentrations

The *Candida* strains were inoculated in Sabouraud Dextrose liquid medium (Biocorp, Warsaw, Poland) and incubated at 37 °C with vigorous shaking (200 rpm) for 24 h before performing the Minimal Inhibitory Concentration (MIC) test. MIC was determined with the microbroth dilution method as recommended by CLSI, with some modifications [30]. Microbial cell suspensions at initial inoculums of 3 × 10^3^ colony forming units per mL in RPMI-1640 medium (with 1-glutamine and phenol red, without bicarbonate) (Sigma-Aldrich, Saint Louis, MO, USA) buffered with 0.165 M 3-(*N*-morpholino)propane sulfonic acid (MOPS) (Sigma-Aldrich, Saint Louis, MO, USA) were exposed to the tested compounds at adequate concentrations (range: 0.001–4 mg/mL for 4-AN, 0.015–10 µg/mL for caspofungin, and 0.15–100 µg/mL for ketoconazole) at 37 °C for 48 h. MIC was the lowest concentration of the compound that inhibited the visible growth of the microorganism. Each experiment was performed twice, and every point was performed in triplicate. The results obtained in each replicate were always identical, thus there are no error bars as ± SD included in the graph presenting MIC values.

### 3.4. In Vitro Biofilm Formation Assay

Biofilm assays to analyze the effect of 4-AN on biofilm development and mature biofilm were carried out following the method reported earlier [31]. The compound was tested at concentrations corresponding to the values of MIC/2, MIC/4, MIC/8, MIC/16, and 1% farnesol was used as a positive control [32].

### 3.5. The Effect of 4-AN on Membrane Permeability

Alteration of membrane permeability was investigated by a crystal violet assay [18,33]. The tested compound corresponding to the concentrations of MIC, 2×MIC, or 4×MIC was added to the cell suspension and incubated at 37 °C, 200 rpm for 8 h. The cells were harvested and 1 × 10^8^ cells per 1 mL were resuspended in 0.85% NaCl solution containing 10 μg/mL of crystal violet. The cell suspensions were incubated at 37 °C, 200 rpm for 10 min. The cells were precipitated by centrifugation at 12,000× *g* at 4 °C for 15 min, and the amount of crystal violet remaining in the supernatant was measured at 590 nm using a spectrophotometer. The OD values of the initial solution of crystal violet used in the assay were regarded as 100%. The percentage of crystal violet uptake of all the cells was calculated as follows: Uptake of crystal violet (%) = (A_590_ of the sample)/(A_590_ of crystal violet solution) × 100.

### 3.6. Sorbitol Protection Assay

The MICs for 4-AN were determined in the presence and absence of 0.8 M sorbitol as an osmotic protectant [19,34]. The *C. albicans* cell suspensions were incubated at 37 °C, and MICs were determined with the same method as described above at 24 h and 72 h.

### 3.7. Atomic Force Microscopy (AFM) Analysis of C. albicans Cells Treated with 4-AN

5 mL of a *Candida albicans* cell suspension cultured under the pressure of amphotericin-B and 4-AN at the concentration of MIC/10 (0.2 µg/mL) and MIC/4 (1 µg/mL), respectively, (DMSO was used as a control) in liquid medium were centrifuged at 8 min 2000× *g*. The supernatant was removed from the precipitate and 5 mL of distilled water were added to the tube and centrifuged for 8 min at 2000× *g*. After centrifugation, the supernatant was removed, and 3 mL of distilled water was added. The slurry was applied to degreased glass slides (10 μL) and allowed to dry at room temperature. The analysis of the topography and sample properties was performed using atomic force microscopy (AFM) Ntegra Spectra C from NT MDT as previously described [6]. The analysis of the obtained data was carried out using NOVA 1.1.0.1824 software (NT-MDT Spectrum Instruments, Moscow, Russia). Topography measurements, including error signal and phase contrast, were performed in a semi-contact mode (Tapping Mode) with a 135 μm NT-MDT NSG03 cantilever with typical resonant frequency 90 kHz and force constant 1.74 N/m. Scanning was performed at 0.5 Hz at a resolution of 512 × 512 pixels. The contact topography measurements with the error signal and lateral force (LF) were made using NT-MDT CSG30 tip lengths of 190 μm and typical force constant of 0.6 N/m. Images were acquired at 0.5 Hz at 512 × 512 pixels. 

Statistical analysis was performed with the use of the STATISTICA v.10 program (StatSoft, Kraków, Polska). The arithmetic means and standard deviations of the parameters analyzed were calculated. Student’s *t*-test for independent samples was used to detect statistically significant differences. The level of significance of *p* < 0.05 was adopted.

### 3.8. Protein Kinase Profiling Assay 

The following protein kinases were tested: HsCDK1/CyclinB1 (human), HsCDK2/CyclinA (human), HsCDK5/p25 (human), HsCDK9/CyclinT (human), HsCK1ε (human), HsGSK3α and HsGSK3β (human), HsHaspin-kd (the kinase domain of the human enzyme), HsPim-1 (human), HsRIPK3 (human), HsAURKB (human Aurora B kinase), LdTLK (Leishmania donovani), LmCK1 (Leishmania major), MmCLK1 (Mus musculus), PfGSK3 (Plasmodium falciparum), RnDYRK1A-kd (Rattus norvegicus), SscCK1δ/ε (porcine), and SscGSK-3α/β (porcine). Kinase enzymatic activities were assayed in 384-well plates using the ADP-GloTM assay kit (Promega, Madison, WI, USA). This assay provides a homogeneous and high-throughput screening method to measure kinase activity by quantifying the amount of ADP produced during a kinase reaction. Briefly, the reactions were carried out in a final volume of 6 µl for 30 min at 30 °C in appropriate kinase buffer, with either protein or peptide as a substrate in the presence of 10 µM ATP. After that, 6 µl of ADP-GloTM kinase reagent were added to stop the kinase reaction. After 50 min incubation at room temperature (RT), 12 µL of kinase detection reagent was added for one hour at RT. The transmitted signal was measured using an Envision (PerkinElmer, Waltham, MA, USA) microplate luminometer and expressed in relative light units (RLU). Kinase activities are expressed in % of the maximal activity, i.e., measured in the absence of the inhibitor. Peptide substrates were obtained from ProteoGenix (Schiltigheim, France). 

### 3.9. Protein Kinase Yak1 3D Model Construction

Template search with BLAST and HHBlits was performed against the SWISS-MODEL template library (SMTL, last update: 12 September 2018, last included PDB release: 7 September 2018). The target sequence was searched with BLAST against the primary amino acid sequence contained in the SMTL. A total of 1063 templates were found. An initial HHblits profile was built using the procedure outlined in Remmert et al. [35], followed by one iteration of HHblits against NR20. The obtained profile was then searched against all profiles of the SMTL. A total of 3719 templates were found. For each identified template, the template quality was predicted from features of the target-template alignment. The templates with the highest quality were then selected for construction of the model. Models were built based on the target-template alignment using ProMod3. Coordinates conserved between the target and the template were copied from the template to the model. Insertions and deletions were remodeled using a fragment library. Side chains were then rebuilt. Finally, the geometry of the resulting model was regularized using a force field. When the loop modeling with ProMod3 failed, an alternative model was built with PROMOD-II [36]. The global and per-residue model quality was assessed using the QMEAN scoring function [37]. For improved performance, the weights of the individual QMEAN terms were trained specifically for the SWISS-MODEL. Ligands present in the template structure were transferred by homology to the model when the following criteria were met: (a) The ligands were annotated as biologically relevant in the template library, (b) the ligand was in contact with the model, (c) the ligand did not clash with the protein, and (d) the residues in contact with the ligand were conserved between the target and the template. If any of these four criteria were not satisfied, the ligand was not included in the model. The model summary includes information on why and which ligand has not been included. The quaternary structure annotation of the template was used to model the target sequence in its oligomeric form. The method is based on a supervised machine learning algorithm, Support Vector Machines (SVM), which combines interface conservation, structural clustering, and other template features to provide a quaternary structure quality estimate (QSQE) [38]. The QSQE score is a number between 0 and 1, reflecting the expected accuracy of the interchain contacts for a model built based on a given alignment and template. Higher numbers indicate higher reliability. This complements the GMQE score, which estimates the accuracy of the tertiary structure of the resulting model.

### 3.10. Molecular Docking Studies

For molecular docking of 4-AN, we selected the X-ray structure of protein kinase DYRK1A from the human (PDB ID: 2WO6) and modeled 3D structure of Yak1 from *C. albicans* as target proteins for the initial docking studies. All Mg^2+^ ions were removed as well as all sulfates, co-solvents, water molecules, and original ligands. The structures were then minimized using the YASARA Energy Minimization Server [39]. Autodock Tools v1.5.6 (The Scripps Research Institute, La Jolla, CA, USA) was used for charging the proteins as well as ligands. Docking calculations were performed with Autodock Vina v1.1.2 (The Scripps Research Institute) under default conditions [40]. During the docking calculations, all the protein residues were fixed and only the inhibitor atoms remained flexible. Visualization of the binding site complexed with the docked ligand was performed by Maestro Suite and PyMOL (Schrödinger) software (v1.2, New York, NY, USA).

## 4. Conclusions

The research revealed the additional antifungal properties of 4-AN. It effectively kills clinical isolates of *Candida*, affects mature biofilm, and moderately disrupts membrane permeability. Interestingly, 4-AN disturbs the growth of the fungal clinical isolates with developed resistance towards the two systemic antifungal drugs, ketoconazole, and caspofungin.

Protein kinases regulate many cellular processes, and their engagement in *Candida* virulence is well documented. In the report, the anti-*Candida albicans* activity of 4-AN is merged with the ability of the compound to inhibit various protein kinases. Our hypothesis is strongly supported with molecular docking studies, which revealed the ability of 4-AN to fit in the ATP-binding pocket of kinases, forming key interactions that are characteristic for the interface of kinase-inhibitor complexes. The presented studies fulfill the growing need for novel therapies in the fight against fungal infections.
